# YTHDF1 promotes breast cancer cell growth, DNA damage repair and chemoresistance

**DOI:** 10.1038/s41419-022-04672-5

**Published:** 2022-03-12

**Authors:** Yu Sun, Dan Dong, Yuhong Xia, Liying Hao, Wei Wang, Chenghai Zhao

**Affiliations:** 1 Department of Pathophysiology, College of Basic Medical Science, China Medical University, Shenyang, China; 2 Department of Pharmaceutical Toxicology, School of Pharmacy, China Medical University, Shenyang, China

**Keywords:** Breast cancer, Breast cancer

## Abstract

Chemoresistance represents a major obstacle to the treatment of human cancers. Increased DNA repair capacity is one of the important mechanisms underlying chemoresistance. *In silico* analysis indicated that YTHDF1, an m6A binding protein, is a putative tumor promoter in breast cancer. Loss of function studies further showed that YTHDF1 promotes breast cancer cell growth in vitro and in vivo. YTHDF1 facilitates S-phase entry, DNA replication and DNA damage repair, and accordingly YTHDF1 knockdown sensitizes breast cancer cells to Adriamycin and Cisplatin as well as Olaparib, a PARP inhibitor. E2F8 is a target molecule by YTHDF1 which modulates E2F8 mRNA stability and DNA damage repair in a METTL14-dependent manner. These data demonstrate that YTHDF1 has a tumor-promoting role in breast cancer, and is a novel target to overcome chemoresistance.

## Introduction

Chemoresistance remains one of the major hurdles in the treatment of human malignant tumors including breast cancer. Enhanced DNA damage repair capacity endues tumor cells with resistance to chemotherapy and radiotherapy. Actually, DNA damage response (DDR) is employed by normal cells to maintain genomic integrity, and defect in DNA damage repair may lead to genomic instability and malignant transformation. In tumor cells, however, DDR deficiency or repression can be utilized to increase the sensitivity to DNA-damaging drugs. Therefore, targeting factors involved in DNA damage repair is an important strategy to overcome chemoresistance and radioresistance [1].

Double-strand breaks (DSBs) are the most serious DNA lesion. They are mainly repaired by homologous recombination (HR) and non-homologous end joining (NHEJ) [2, 3]. Factors such as RAD51, BRCA1, BRCA2, BARD1, and PALB2 are critical for DSB HR repair [4]. Among them, both RAD51 and BRCA1 contribute to the resistance to Adriamycin and Cisplatin in breast cancer [5–7]. Moreover, RAD51 plays crucial roles in the resistance to PARP inhibitor Olaparib [8].

N6-methyladenosine (m6A) is the most common eukaryotic mRNA modification. Methyltransferases such as METTL3 and METTL14, install methyl group on the N6-position of adenosine. Some “reader” proteins can recognize and bind m6A site, modulating RNA metabolism including splicing, export, degradation, and translation [9, 10]. YTHDF1 is an m6A binding protein involved in tumorigenesis and metastasis through modulating translation and stability of target mRNAs [11–16].

Here a novel function of YTHDF1 in human tumors was identified. YTHDF1 promotes DNA damage repair, especially DSBs HR repair, in a METTL14-dependent manner. Accordingly, YTHDF1 induces the resistance to DNA-damaging drugs Adriamycin and Cisplatin, and PARP inhibitor Olaparib.

## Results

### *In silico* analysis indicates YTHDF1 as a potential tumor promoter in breast cancer

**Fig. 1:**
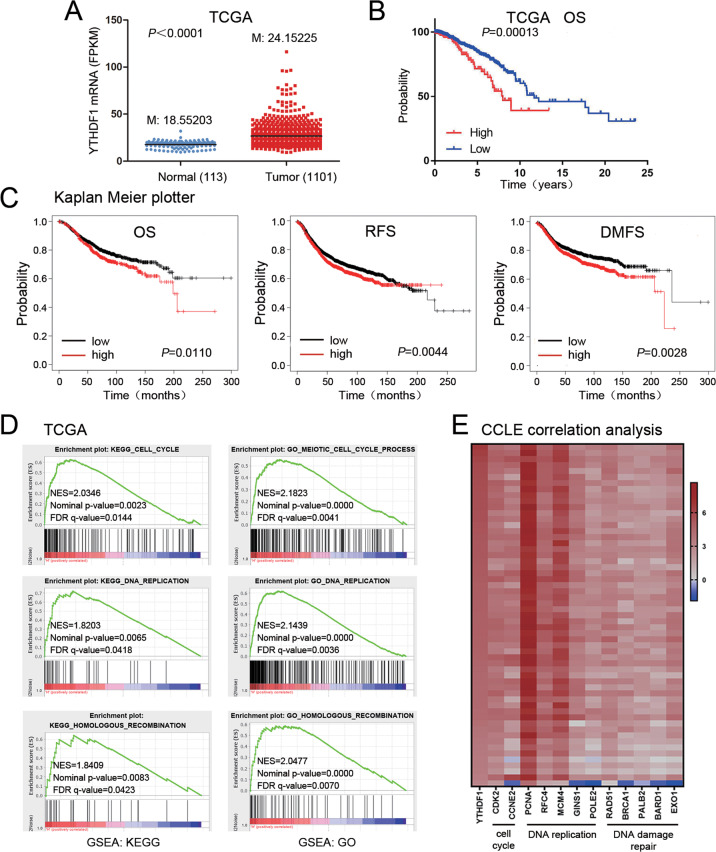
*In silico* analysis indicates YTHDF1 as a potential tumor promoter in breast cancer. **A** YTHDF1 mRNA expression was investigated in TCGA database and compared between breast cancer samples (*n* = 1101) and normal mammary tissues (*n* = 113). M: median value. **B** The association of YTHDF1 with OS was analyzed in TCGA database. **C** The association of YTHDF1 with OS, RFS and DMFS was analyzed in Kaplan Meier plotter website. **D** GSEA in TCGA database was performed. YTHDF1-related enrichment plots were shown. **E** A heat map was generated from CCLE database indicating the correlation of YTHDF1 with factors related to cell cycle, DNA replication and DNA damage repair.

Databases were used to analyze the potential role of YTHDF1 in breast cancer. In TCGA database, cancer tissues expressed higher level of YTHDF1 compared to normal tissues (Fig. 1A). In Curtis database, YTHDF1 was overexpressed in invasive ductal carcinoma, invasive lobular carcinoma, mucinous carcinoma, and medullary carcinoma, but not in benign breast neoplasm (Supplementary Fig. 1). Higher YTHDF1 level was correlated with shorter overall survival (OS), recurrence-free survival (RFS), and distal metastasis-free survival (DMFS) in the TCGA database and Kaplan Meier plotter website (Fig. 1B, C). GSEA in TCGA database indicated a relation of YTHDF1 to cell cycle, DNA replication and DNA damage repair (Fig. 1D). Furthermore, GSEA revealed a relation of YTHDF1 to mRNA export and processing (Supplementary Fig. 2). Finally, YTHDF1 was positively correlated with factors related to cell cycle, DNA replication and DNA damage repair in CCLE database (Fig. 1E). These findings indicated that YTHDF1 functioned as a putative tumor promoter in breast cancer.

### YTHDF1 induces breast cancer cell growth in vitro and in vivo

**Fig. 2:**
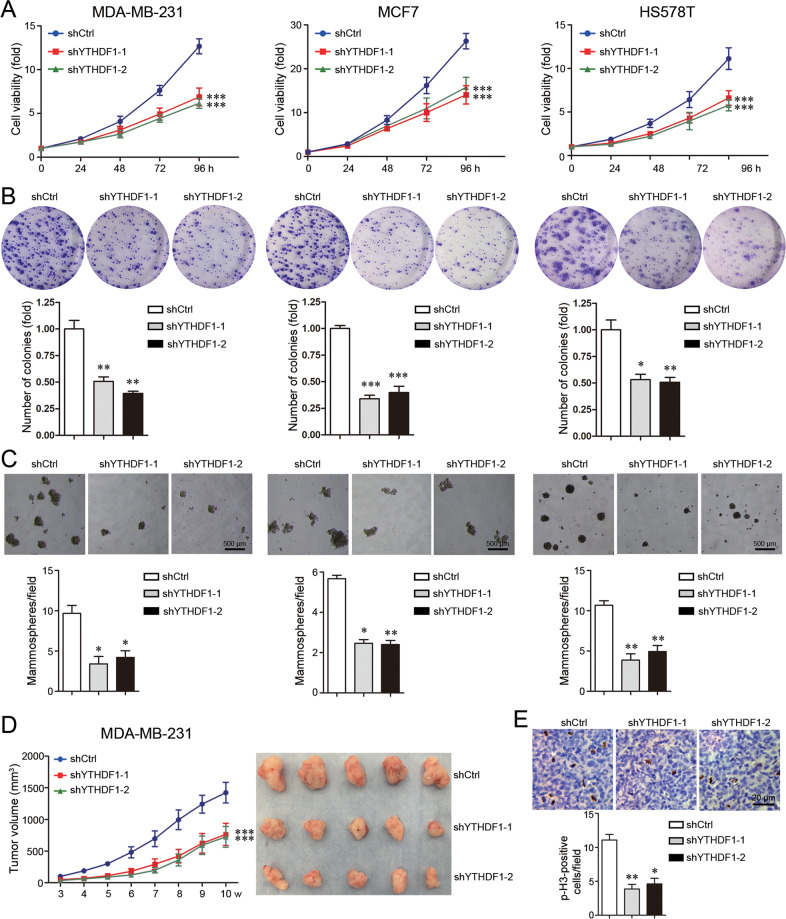
YTHDF1 induces cell growth in vitro and in vivo. **A** Cell viability was analyzed by CCK8. Mean±SD, *n* = 3. ***P < 0.001, vs shCtrl. **B** Cell growth was determined by Colony formation. Mean±SD, *n* = 3. *P < 0.05, **P < 0.01, ***P < 0.001, *vs* shCtrl. **C** Mammosphere formation was shown. Scale bar: 500μm. Mean±SD, *n* = 3. *P < 0.05, **P < 0.01, vs shCtrl. **D** Xenograft tumors from MDA-MB-231 cells and their growth curves were shown. Mean±SD, *n* = 5. ***P < 0.001, vs shCtrl. (E) Expression of p-H3 in xenograft tumors was detected by Immunohistochemistry. Scale bar: 20 μm. Mean±SD, *n* = 3. *P < 0.05, **P < 0.01, vs shCtrl.

To verify the tumor-promoting role of YTHDF1, breast cancer cell lines MDA-MB-231, MCF7 and HS578T were selected to stably knockdown YTHDF1 expression (Supplementary Fig. 3, Supplementary file 1). As shown by CCK8 and Colony formation tests, YTHDF1 knockdown remarkably suppressed cell growth in vitro (Fig. 2A, B). To the contrary, YTHDF1 overexpression promoted cell growth (Supplementary Fig. 4). YTHDF1 knockdown interfered with mammosphere formation, indicating that YTHDF1 contributed to the tumorigenic growth (Fig. 2C). MDA-MB-231 cells were inoculated into immune-deficient mice to evaluate whether YTHDF1 affects cell growth in vivo. Consistently, YTHDF1 knockdown impeded tumor growth significantly (Fig. 2D). Tumors with YTHDF1 knockdown had less cells with positive staining of phosphorylated Histone 3 (p-H3), a factor reflecting cell division, compared to those with control knockdown (Fig. 2E).

### YTHDF1 promotes S-phase entry and DNA replication

**Fig. 3:**
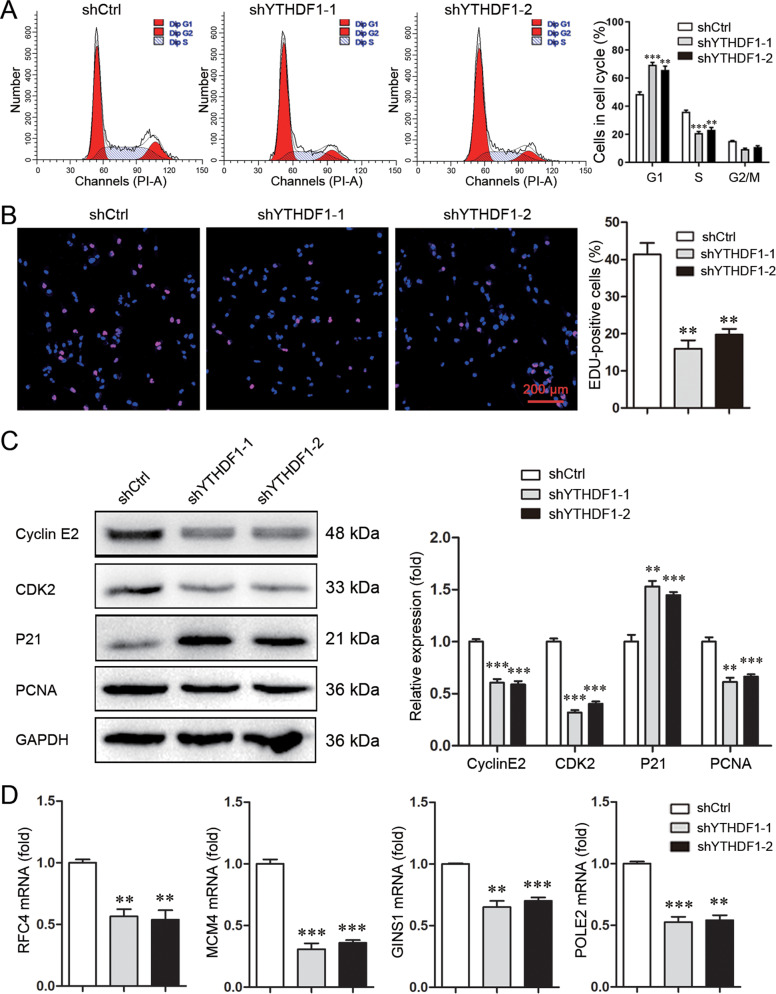
YTHDF1 promotes S-phase entry and DNA replication. **A** Cell cycle of MDA-MB-231 cells was analyzed by Flowcytometry. Mean±SD, *n* = 3. **P < 0.01, ***P < 0.001, *vs* shCtrl. **B** DNA replication of MDA-MB-231 cells was determined by EDU staining. Scale bar: 200 μm. Mean±SD, *n* = 3. **P < 0.01, vs. shCtrl. **C** Cyclin E2, CDK2, P21 and PCNA expression in MDA-MB-231 cells was detected by Western blot. Mean±SD, *n* = 3. **P < 0.01, ***P < 0.001, vs. shCtrl. **D** RFC4, MCM4, GINS1 and POLE2 expression in MDA-MB-231 cells was detected by Real-time PCR. Mean±SD, *n* = 3. **P < 0.01, ***P < 0.001, vs. shCtrl.

Based on the *in silico* analysis, the effect of YTHDF1 on the cell cycle and DNA replication was investigated. YTHDF1 knockdown in MDA-MB-231 cells reduced S-phase fraction while increased G1-phase fraction (Fig. 3A). This finding was supported by EDU staining. YTHDF1 knockdown diminished the fraction of EDU-positive cells (Fig. 3B). Mechanistically, YTHDF1 knockdown downregulated CDK2 and Cyclin E2 whereas upregulated P21, a CDK inhibitor involved in S-phase (Fig. 3C, Supplementary file 1). YTHDF1 knockdown downregulated a series of factors related to DNA replication including PCNA, RFC4, MCM4, GINS1, and POLE2 (Fig. 3C, D). Similar findings were observed in YTHDF1-knockdowned MCF7 cells (Supplementary Fig. 5, Supplementary file 1). These data indicated that YTHDF1 promoted S-phase entry and DNA replication.

### YTHDF1 enhances DNA damage repair and chemoresistance

**Fig. 4:**
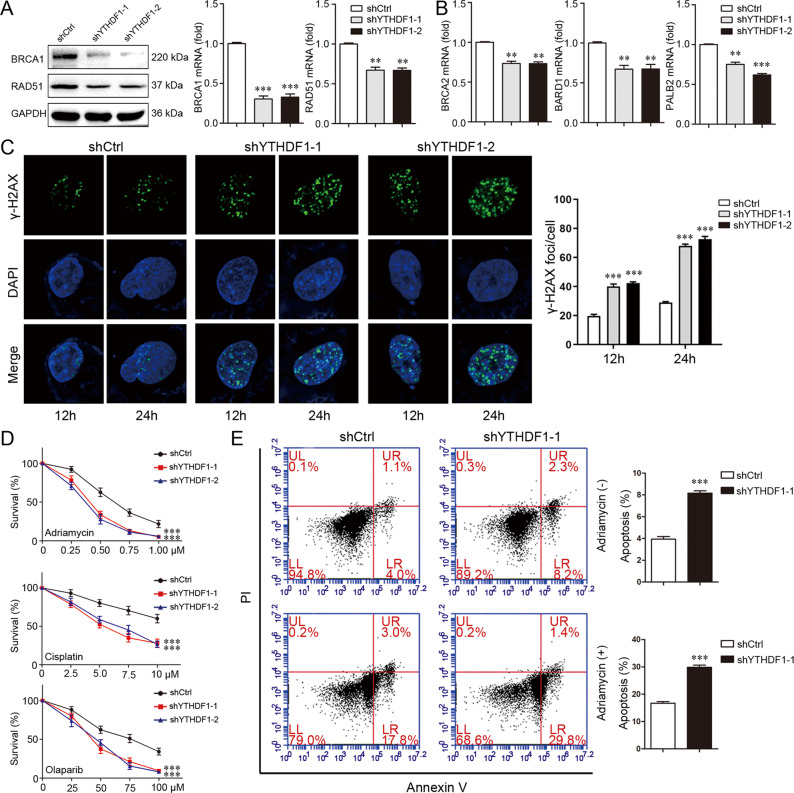
YTHDF1 enhances DNA damage repair and chemoresistance. **A** BRCA1 and RAD51 expression in MDA-MB-231 cells was detected by Western blot and real-time PCR. Mean±SD, *n* = 3. **P < 0.01, ***P < 0.001, vs shCtrl. **B** BRCA2, BARD1 and PALB2 expression in MDA-MB-231 cells was detected by real-time PCR. Mean±SD, *n* = 3. **P < 0.01, ***P < 0.001, vs shCtrl. **C** γ-H2AX foci formation in MDA-MB-231 cells was detected by Immunofluorescence 12 or 24 h after treatment with Adriamycin (300 nM). Mean±SD, *n* = 3. ***P < 0.001, vs shCtrl. **D** Viability of MDA-MB-231 cells was analyzed by CCK8 48 h after treatment with different concentrations of Adriamycin, Cisplatin or Olaparib. Mean±SD, *n* = 3. ***P < 0.001, vs shCtrl. (E) Death of MDA-MB-231 cells was detected by Flowcytometry 24 h after treatment with or without Adriamycin (300 nM). Mean±SD, *n* = 3. ***P < 0.001, vs shCtrl.

The role of YTHDF1 in DNA damage repair and chemoresistance was subsequently explored. YTHDF1 knockdown in MDA-MB-231 cells downregulated BRCA1 and RAD51 in both mRNA and protein levels (Fig. 4A, Supplementary file 1). Moreover, YTHDF1 knockdown downregulated HR-related factors BRCA2, BARD1, and PALB2 (Fig. 4B). Adriamycin was used to induce DNA damage including DNA DSBs by inhibiting DNA topoisomerase II. 12 or 24 h after treatment with Adriamycin, cells with YTHDF1 knockdown exhibited more γ-H2AX foci compared to cells with control knockdown, demonstrating an impaired repair capacity to Adriamycin-induced DNA damage (Fig. 4C). Moreover, YTHDF1 knockdown impaired RAD51 recruitment to DNA damage site (Supplementary Fig. 6). YTHDF1 knockdown increased the sensitivity of breast cancer cells to Adriamycin, Cisplatin, and Olaparib (Fig. 4D) and promoted Adriamycin-induced cell apoptosis (Fig. 4E), indicating that YTHDF1 contributed to chemoresistance. Similarly, YTHDF1 knockdown repressed DNA damage repair and chemoresistance in MCF7 cells (Supplementary Fig. 7, Supplementary file 1). Finally, YTHDF1 overexpression in MDA-MB-231 cells promoted DNA damage repair and chemoresistance (Supplementary Fig. 8).

### E2F8 is a target molecule by YTHDF1

**Fig. 5:**
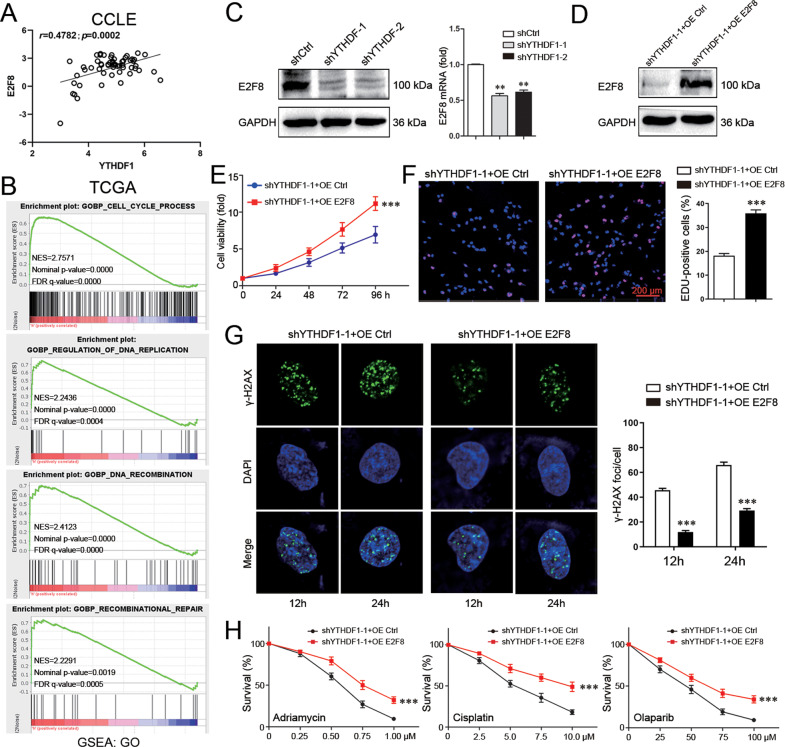
E2F8 is a target molecule by YTHDF1. **A** CCLE database was interrogated for YTHDF1 and E2F8 expression. Correlation between YTHDF1 and E2F8 was analyzed by Pearson statistics. **B** GSEA in TCGA database was performed. E2F8-related enrichment plots were shown. **C** E2F8 expression in MDA-MB-231 cells was detected by Western blot and Real-time PCR. Mean±SD, *n* = 3. **P < 0.01, vs shCtrl. **D** E2F8 expression in MDA-MB-231 cells was detected by Western blot. **E** Viability of MDA-MB-231 cells was analyzed by CCK8. Mean±SD, *n* = 3. ***P < 0.001, vs shYTHDF1-1+OE Ctrl. **F** DNA replication of MDA-MB-231 cells was determined by EDU staining. Scale bar: 200 μm. Mean±SD, *n* = 3. ***P < 0.001, vs shYTHDF1-1+OE Ctrl. **G** γ-H2AX foci formation in MDA-MB-231 cells was detected by Immunofluorescence 12 or 24 h after treatment with Adriamycin (300 nM). Mean±SD, *n* = 3. ***P < 0.001, vs shYTHDF1-1+OE Ctrl. **H** Viability of MDA-MB-231 cells was analyzed by CCK8 48 h after treatment with different concentrations of Adriamycin, Cisplatin or Olaparib. Mean±SD, *n* = 3. ***P < 0.001, vs shYTHDF1-1+OE Ctrl.

CCLE database was investigated to search the putative downstream component of YTHDF1. E2F8 was identified to be highly correlated with YTHDF1 (Fig. 5A). GSEA in TCGA database consistently revealed that E2F8 was related to not only cell cycle and DNA replication, but also DNA damage repair (Fig. 5B). YTHDF1 knockdown in MDA-MB-231 cells downregulated E2F8 in both mRNA and protein levels, indicating E2F8 as a downstream target of YTHDF1 (Fig. 5C, Supplementary file 1). Furthermore, E2F8 overexpression in YTHDF1-knockdowned MDA-MB-231 cells rescued YTHDF1-associated phenotype (Fig. 5D, Supplementary file 1). Specifically, E2F8 overexpression increased cell growth, elevated the fraction of EDU-positive cells, attenuated γ-H2AX staining after Adriamycin treatment, and reduced the sensitivity to Adriamycin, Cisplatin, and Olaparib (Fig. 5E–H, Supplementary Fig. 9A). These findings demonstrated that E2F8 at least in part mediated YTHDF1-induced DNA replication, DNA damage repair, and chemoresistance.

### YTHDF1 enhances E2F8 mRNA stability dependent on METTL14

**Fig. 6:**
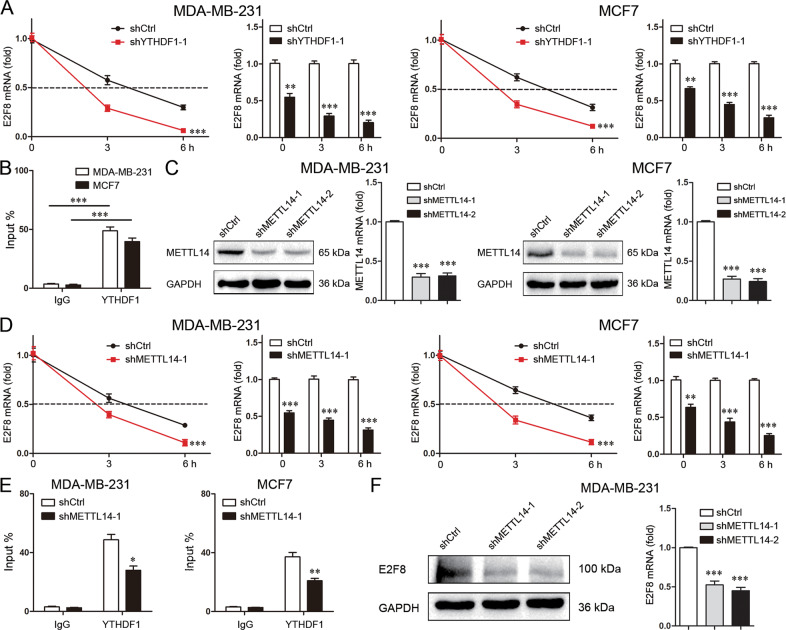
YTHDF1 enhances E2F8 mRNA stability dependent on METTL14. **A** E2F8 mRNA level was detected by real-time PCR in MDA-MB-231 and MCF7 cells after treatment of Actinomycin D (5 μg/ml). Mean±SD, *n* = 3. **P < 0.01, ***P < 0.001, vs shCtrl. **B** Binding of YTHDF1 to E2F8 mRNA in MDA-MB-231 and MCF7 cells was analyzed by RIP and real-time PCR. Mean±SD, *n* = 3. ***P < 0.001. **C** METTL14 expression was detected by Western blot and real-time PCR in MDA-MB-231 and MCF7 cells. Mean±SD, *n* = 3. ***P < 0.001, vs shCtrl. **D** E2F8 mRNA level was detected by real-time PCR in MDA-MB-231 and MCF7 cells after treatment of Actinomycin D (5 μg/ml). Mean±SD, *n* = 3. **P < 0.01, ***P < 0.001, vs shCtrl. **E** Binding of YTHDF1 to E2F8 mRNA in MDA-MB-231 and MCF7 cells was analyzed by RIP and real-time PCR. Mean±SD, n = 3. *P < 0.05, **P < 0.01, vs shCtrl. **F** E2F8 expression was detected by Western blot and real-time PCR in MDA-MB-231 cells. Mean±SD, *n* = 3. ***P < 0.001, vs shCtrl.

As an m6A “reader”, the effect of YTHDF1 on E2F8 mRNA stability was evaluated. As expected, YTHDF1 knockdown significantly shortened E2F8 mRNA half-lives (Fig. 6A). RIP test further confirmed the binding of YTHDF1 to E2F8 mRNA in the predicted m6A sites (Fig. 6B). To investigate the involvement of METTL14 in this process, METTL14 was stably knockdowned in breast cancer cells (Fig. 6C, Supplementary file 1). As shown in Fig. 6D, METTL14 knockdown similarly reduced E2F8 mRNA stability. METTL14 knockdown interfered with the binding of YTHDF1 to E2F8 mRNA (Fig. 6E), and suppressed E2F8 expression in both mRNA and protein levels (Fig. 6F, Supplementary file 1). These data indicated that YTHDF1 impeded E2F8 mRNA decay dependent on METTL14.

### METTL14 is involved in DNA replication, DNA damage repair and chemoresistance

**Fig. 7:**
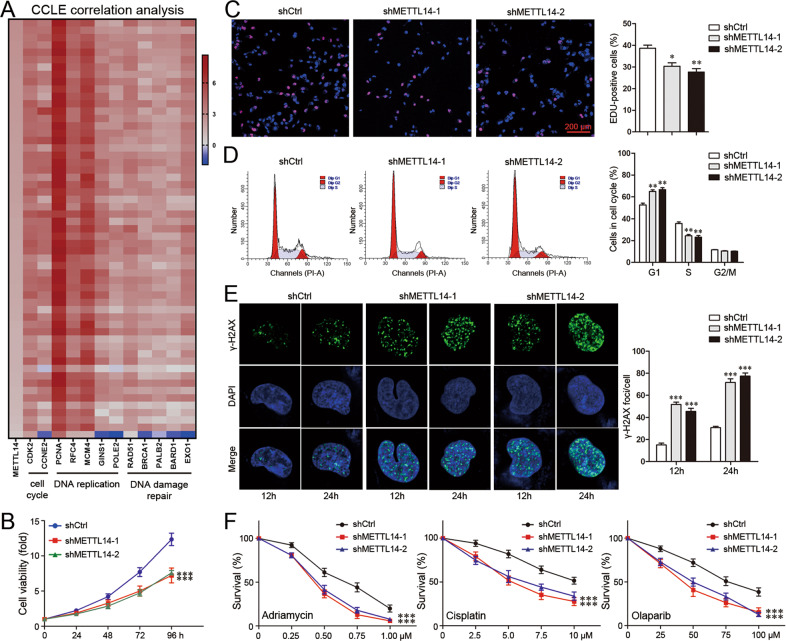
METTL14 is involved in DNA replication, DNA damage repair and chemoresistance. **A** A heat map was generated from CCLE database indicating the correlation of METTL14 with factors related to cell cycle, DNA replication and DNA damage repair. **B** Viability of MDA-MB-231 cells was analyzed by CCK8. Mean±SD, *n* = 3. ***P < 0.001, vs shCtrl. **C** DNA replication of MDA-MB-231 cells was determined by EDU staining. Scale bar: 200 μm. Mean±SD, *n* = 3. *P < 0.05, **P < 0.01, vs shCtrl. **D** Cell cycle of MDA-MB-231 cells was analyzed by Flowcytometry. Mean±SD, *n* = 3. **P < 0.01, vs shCtrl. **E** γ-H2AX foci formation in MDA-MB-231 cells was detected by Immunofluorescence 12 or 24 h after treatment with Adriamycin (300 nM). Mean±SD, *n* = 3. ***P < 0.001, vs shCtrl. **F** Viability of MDA-MB-231 cells was analyzed by CCK8 48 h after treatment with different concentrations of Adriamycin, Cisplatin, or Olaparib. Mean±SD, *n* = 3. ***P < 0.001, vs shCtrl.

As METTL14 coordinated with YTHDF1 to modulate E2F8, the involvement of METTL14 in YTHDF1-associated phenotypes was further clarified. METTL14 was positively correlated with factors related to cell cycle, DNA replication and DNA damage repair in the CCLE database (Fig. 7A). Consistently, METTL14 knockdown in MDA-MB-231 cells reduced cell growth (Fig. 7B, Supplementary Fig. 9B) and repressed DNA replication (Fig. 7C, D). METTL14-knockdowned cells exhibited more γ-H2AX staining after Adriamycin treatment, indicating that METTL14 knockdown interfered with DNA damage repair (Fig. 7E). As a consequence, METTL14 knockdown enhanced the sensitivity to Adriamycin, Cisplatin, and Olaparib (Fig. 7F). These data demonstrated that METTL14 promoted DNA replication, DNA damage repair, and chemoresistance.

## Discussion

Several lines of evidence indicate that YTHDF1 functions as a putative tumor-promoter in breast cancer. First, YTHDF1 is overexpressed in breast cancer tissues. Second, higher YTHDF1 expression is correlated with shorter survival. Third, YTHDF1 promotes breast cancer cell growth in vitro and in vivo. YTHDF1 facilitate S-phase entry and DNA replication through modulating a series of factors related to S-phase, including Cyclin E2, CDK2, P21 and PCNA. Notably, DNA replication is closely associated with DNA damage repair, and HR is active in S-phase of the cell cycle [17, 18].

The link between DNA damage repair and m6A modification has been revealed by several recent studies. METTL3 is involved in nucleotide excision repair of UV-induced DNA damage [19]. Moreover, METTL3 and m6A binding protein YTHDC1 are responsible for DSBs HR repair in human sarcoma U2OS cells; METTL3 depletion enhances the sensitivity to DNA-damaging therapy [20]. The finding that YTHDF1 contributes to HR repair and chemoresistance in breast cancer further confirms the association of m6A modification with DNA damage repair. Mechanistically, YTHDF1 upregulates HR-related factors RAD51 and BRCA1, as well as BRCA2, BARD1, and PALB2 in a METTL14-dependent manner.

PARP inhibition is synthetic lethal with DSBs HR deficiency. Currently, PARP inhibitors are prescribed to patients with germline BRCA1/2 mutations [21]. In tumors with other HR defects such as germline PALB2 or somatic BRCA1/2 mutations, Olaparib is also effective [22]. However, PARP inhibitors are less effective on HR-proficient tumors. YTHDF1 knockdown sensitizes HR-proficient cells to Olaparib, mainly due to the suppression of HR repair. Clearly, targeting HR repair is a potential strategy to extend the use of PARP inhibitors to HR-proficient tumors [23–25].

E2F8 functions as a tumor promoter in breast cancer by promoting cell proliferation [26, 27]. As a downstream target of YTHDF1-METTL14, E2F8 was first identified to be involved in DNA damage repair and chemoresistance in breast cancer. Actually, the role of E2F8 in DNA damage repair remains largely unknown in human tumors. In a study using U2OS cells, E2F8 is shown to be required for the cell-cycle response to DNA damage [28]. Unlike E2F8, E2F1, another member in the E2F family, has been demonstrated to play an important role in DNA damage repair [29].

In summary, YTHDF1 for the first time was uncovered to promote S-phase entry, DNA replication and DNA damage repair. YTHDF1 is not only a tumor promoter but also a target to overcome chemoresistance in breast cancer. Furthermore, the role of YTHDF1 in DNA replication and repair is dependent on METTL14 and mediated by E2F8.

## Materials and methods

### *In silico* analysis

The Cancer Genome Atlas (TCGA) database was interrogated for YTHDF1 mRNA expression in breast cancer samples (*n* = 1101) and normal mammary tissues (*n* = 113). YTHDF1 expression in Curtis database was investigated in various breast tumors and normal mammary tissues in Oncomine (https://www.oncomine.org/). The correlation of YTHDF1 with survival was analyzed in the TCGA database and Kaplan-Meier plotter website (http://kmplot.com/analysis/). GSEA was conducted in TCGA database. The samples were divided into high and low groups according to gene expression. Gene pathways differentially expressed between high and low groups were analyzed. The Cancer Cell Line Encyclopedia (CCLE) database was interrogated for gene expression in human breast cancer cell lines. Correlation between two genes was analyzed by Pearson statistics.

### Cell culture

MDA-MB-231, MCF7, and HS578T cells were obtained from Nanjing KeyGen Biology (Nanjing, China). All human cell lines have been authenticated using STR profiling. These cells were cultured in DMED (Hyclone) with 10% fetal bovine serum (FBS) at 37 °C in a humidified incubator with 5% CO_2_.

### Cell transfection

MDA-MB-231, MCF7, and HS578T cells were transfected with shYTHDF1 lentiviruses (GV112/hU6-MCS-CMV-Puromycin, Genechem, China) to stably knockdown YTHDF1 expression. MDA-MB-231 and MCF7 cells were transfected with shMETTL14 lentiviruses (BSR-LW012/Plv-U6-shRNA-EF1α-Puromycin, SyngenTech, China) to stably knockdown METTL14 expression. After infection for 72 h, cells were selected by 2 μg/ml puromycin (Sigma). MDA-MB-231 cell was transfected with E2F8 overexpression plasmids (GV219/CMV-MCS-SV40-Neomycin, Genechem, China) by Lipofectamine 3000 in Opti-MEM medium according to the product manual. The target sequence for shYTHDF1-1 is 5′- CGCCGTCCATTGGATTTCCTT-3′, for shYTHDF1-2 is 5′- AACCTCCATCTTCGACGACTT-3′, and for control is 5′-TTCTCCGAACGTGTCACGT-3′. The target sequence for shMETTL14-1 is 5′-CCATGTACTTACAAGCCGATA-3′, for shMETTL14-2 is 5′- GCCGTGGACGAGAAAGAAATA -3′, and for control is 5′- CCTAAGGTTAAGTCGCCCTCGC -3′.

### Colony formation assay

Cells (MDA-MB-231 and HS578T: 2×10^3^; MCF7: 6×10^2^) were trypsinized, counted, and cultured in 3.5 cm plates in medium with 10% FBS containing 5% CO_2_ for 2 weeks. Cells were washed with PBS, fixed with 4% paraformaldehyde for 15 min and subsequently stained with 1% crystal violet for 30 min at 37 °C. Colonies were counted and photographed.

### Tumorisphere formation

Cells (MDA-MB-231 and HS578T: 1×10^4^; MCF7: 5×10^3^) were trypsinized, counted, and seeded into six-well low-attachment surface polystyrene culture plates (Corning Costar, USA). Cells were cultured in complete MammoCult™ Human Medium (STEMCELL Technologies, USA) at 37 °C and 5% CO_2_ for 2 weeks. Spheroids in five randomly selected fields were counted.

### Cell viability assay

For proliferation assay, cells were trypsinized, counted, and seeded into 96-well plates (MDA-MB-231: 5×10^3^; MCF7: 5×10^2^; HS578T: 2×10^3^) in 100 μl medium with 10% FBS. 10 μl Cell Counting Kit-8 (Dojindo Molecular Technologies, Japan) was added into each well. Then cells were incubated in a humidified incubator for 1 h at 37 °C with 5% CO_2_. The absorbance was measured at 450 nm by a microplate reader (Bio-Rad Laboratories, USA) at different time points. For cytotoxic assay, cells (MDA-MB-231: 8×10^3^; MCF7: 2×10^3^) were cultured for 24 h. Different concentration of Adriamycin (0, 0.25, 0.5, 0.75, 1 μM), Cisplatin (0, 2.5, 5, 7.5, 10 μM) or Olaparib (0, 25, 50, 75, 100 μM) was added for 48 h.

### In vivo animal study

Female BALB/c nude mice (5~6 weeks of age, 18–20 g) were purchased from Weitong Lihua (Beijing, China). All animals were dealt with according to the Animal Ethics Committee of China Medical University. Before tumor cell inoculation, mice were randomized into different groups (five in each group). 1×10^6^ MDA-MB-231 cells were resuspended in 100 μl PBS with 50% Matrigel (Corning Costar, USA), and injected into the mammary fat pad of the mice. The length and width of tumors were measured with a vernier caliper every week. Tumor volume was calculated by the formula: *V* = 1/2×length×width^2^. The investigator was blinded to the group allocation of the animals during the experiment. No statistical method was used to predetermine the sample size for the xenograft mice experiment, which was based on previous experimental observations. The sample size of each experiment is shown in the legend. No data were excluded from the analysis.

### Immunohistochemistry

The tissues were first fixed in 4% paraformaldehyde for 72 h, and then dehydrated. Next the tissues were embedded in paraffin and then sliced into 4 μm sections. The sections were deparaffinized and hydrated with xylene and gradient alcohol, respectively. Three percentage H_2_O_2_ was used to eliminate endogenous peroxidase activity. The sections were further repaired with citrate buffer, and then blocked by BSA. Subsequently, the sections were incubated with primary antibody (anti-phosphorylated Histone 3, ThermoFisher, PA5-17869, USA, 1:200) overnight at 4 °C, incubated with goat anti-rabbit IgG and streptavidin peroxidase (SP) complex at 37 °C for 30 min, and stained with DAB reagent. At last, the sections were re-stained with hematoxylin, dehydrated with gradient alcohol, mounted, and photographed under a microscope (LEICA DM2500 LED). Phosphorylated Histone 3-positive cells in five randomly selected fields were counted.

### Cell cycle assay

1×10^6^ cells were harvested, washed with PBS, and fixed with 70% alcohol at 4 °C overnight. Then cells were treated with 500ul PI/RNaSeA staining solution (KeyGEN BioTECH, China) for 1 h at room temperature in the dark according to the instructions, and analyzed by a FACS Calibur Flow Cytometer (BD).

### EDU staining

5×10^5^ cells were trypsinized, counted, and seeded into 24-well culture plates for 24 h. After addition of 50 μM EDU (Ribobio, China), cells were incubated for another 2 h. Then cells were washed with PBS, fixed with 4% paraformaldehyde for 30 min and permeabilized with 0.5% Triton X-100 for 10 min. Subsequently, cells were treated with 200 μl 1×Apollo reaction cocktail for 30 min at room temperature in the dark. The DNA contents were stained by 200 μl 1×Hoechst 33342 for 30 min at room temperature in the dark. Cells were observed under a laser scanning confocal focus microscope (FV-1000; Olympus). Positive cells in random five fields were counted.

### Western blot

Cells were lysed by RIPA lysis with 1% PMSF on ice for 1 h, and centrifuged with 12,000× *g* at 4 °C for 40 min. The protein concentration was determined by a BCA protein assay kit (Wanleibio, China). 30 μg protein was separated on 10% SDS-PAGE gel and transferred into a polyvinylidene difluoride (PVDF) membrane in a wet electron transfer device. 5% skimmed milk in Tris-buffered saline (TBS) containing 0.05% Tween 20 was used to block the membrane for 2 h at room temperature. The membranes were incubated with different primary antibodies at 4 °C overnight and incubated with horseradish peroxidase (HRP)-conjugated goat anti-rabbit or goat anti-mouse for 1.5 h at room temperature. The primary antibodies are as follows: YTHDF1 (Cell Signaling Technology, #86463, USA, 1:1000), BRCA1 (Cell Signaling Technology, #9010, USA, 1:1000), CDK2 (Cell Signaling Technology, #2546, USA, 1:1000), Cyclin E2 (Cell Signaling Technology, #4132, USA, 1:1000), PCNA (SANTA, sc-71858, USA, 1:1000), RAD51 (Abcam, ab133534, USA, 1:1000), P21 (Cell Signaling Technology, #2947, USA, 1:1000), E2F8 (Abcam, ab109596, USA, 1:2000), METTL14 (Cell Signaling Technology, #51104, USA, 1:1000). An enhanced chemiluminescene (ECL) kit (Wanleibio, China) was used to visualize the target protein.

### Real-time PCR

Total RNA was extracted from cells with TRIZOL Reagent (Takara, 9108/9109, China) following the standard instructions. Reverse transcription was performed with the cDNA synthesis Kit (Takara, RR047A, China) with 1 μg RNA. The target cDNA was amplified by TB Green™ Premix Ex Taq II and an ABI PRISM 7300 Sequence Detection system (Applied Biosystems, USA). The relative gene expression was analyzed by the 2^−ΔΔCt^ method. GAPDH was used as control. The primers are listed in Table 1.

**Table 1:** Primers for Real-time PCR.

Genes or binding sites	Primers (5′–3′)
YTHDF1-forward	GACGACATCCACCGCTCCATTAAG
YTHDF1-reverse	CCCACTCCCATTGACGCTGAAG
RFC4-forward	AAACCACCCGATTCTGTCTTAT
RFC4-reverse	CTTGGCAATGTCTAGTAATCGC
MCM4-forward	ATCTCCCTCTCAGAGACGTAG
MCM4-reverse	TGTCAGTGGTGAACTAACATCA
METTL14-forward	ACCAAAATCGCCTCCTCCCAAATC
METTL14-reverse	AGCCACCTCTTTCTCCTCGGAAG
E2F8-forward	CAAACCACAGGATTTACAGCTC
E2F8-reverse	CCATTAGCTTCAACGGTGTTAC
GINS1-forward	AGAGCACTCAGATGGGAATATG
GINS1-reverse	ATCCTGTGTAATGTCCAAACCT
POLE2-forward	GTCTTAGCAGAAGGTTGGTTTG
POLE2-reverse	TGCAGAAGTCTTCACAGATGTA
BRCA1-forward	AGGTCCAAAGCGAGCAAGAGAATC
BRCA1-reverse	CTGTGGGCATGTTGGTGAAGGG
BRCA2-forward	GTCTTTCCACAGCCAGGCAGTC
BRCA2-reverse	GAGAACACGCAGAGGGAACTTGG
RAD51-forward	TGGCAGTGGCTGAGAGGTATGG
RAD51-reverse	GGTCTGGTGGTCTGTGTTGAACG
BARD1-forward	TGCTACTTCTATTTGTGGGGAACCTTC
BARD1-reverse	CTGTCTGGCTTGGGCTTTCTACTG
PALB2-forward	AGGGAATACAGCAAGACACTAG
PALB2-reverse	GATCCTGCTGAGACAAACAATC
GAPDH-forward	CAGGAGGCATTGCTGATGAT
GAPDH-reverse	GAAGGCTGGGGCTCATTT

### Immunofluorescence

5×10^5^ cells were trypsinized, counted, and seeded into 24-well culture plates for 24 h. After addition of 300 nM adriamycin, cells were incubated for another 12 h or 24 h. Then cells were washed with PBS and fixed with 4% paraformaldehyde at room temperature for 30 min. 0.5% Triton X-100 was used to permeabilize cells at room temperature for 10 min. Subsequently, cells were blocked with 5% donkey serum in PBS at room temperature for 1 h, and incubated with primary antibody for γ-H2AX (Cell Signaling Technology, #9718, USA, 1:400) at 4 °C overnight and with secondary antibody Alexa Fluor 488-conjugated anti-rabbit IgG (Invitrogen, USA) for 2 h at room temperature in the dark. The nuclei were stained by DAPI (Beyotime, China) at room temperature for 5 min. Immunofluorescence staining was observed under a laser scanning confocal focus microscope (FV-1000; Olympus).

### Apoptosis assay

5×10^5^ cells were harvested and washed with PBS. Following the standard protocol, an Annexin V-FITC/PI Apoptosis Kit (KeyGEN BioTECH, China) was used to analyze cell apoptosis. Cells were incubated in 500 μl binding buffer containing 5 μl Annexin V-FITC and 5 μl PI at room temperature in the dark for 15 min. The number of apoptotic cells was measured by an Accuri C6 Plus Flow Cytometer (BD).

### mRNA stability assay

Cells were harvested and seeded into 6-well culture plates for 24 h. After addition of 5 μg/ml actinomycin D (Sigma, A9415), cells were cultured for 3 h or 6 h, and collected. Total RNA was extracted for reverse transcription and Real-time PCR.

### RNA immunoprecipitation (RIP) assay

5×10^6^ cells were harvested and washed with cold PBS. According to the standard instructions of the Imprint RNA RIP kit (Sigma), cells were lysed with the lysis buffer in −80 °C freezer overnight. 5 μg antibody (anti-YTHDF1: Cell Signaling Technology, #86463, USA, 1:50) was prebound to 20 μl magnetic beads in RIP wash buffer with rotation for 30 min at room temperature, and then the cell lysates were added at 4 °C with rotation overnight. Then RNA was extracted and dissolved with RNase-free water. The enrichment of certain fragments was determined by real-time PCR. Primer used for E2F8 quantification was designed as follows: forward: 5’- AGTGCTTTGTATCTTTAAGGAAGCCC-3’, reverse: 5’-AGCAGTAAAGTCGTGGGAGGT-3’. IgG was used as the negative control.

### Statistical analysis

All cell experiments were performed triplicate. The data are expressed as mean ± SD. GraphPad prism 8 was used to analyze the data. Differences were analyzed by two-sided Student’s *t* test or two-way ANOVA when the variance is similar between the groups. *P* value < 0.05 was considered statistically significant.

## Supplementary information

Supplementary Figure 1

Supplementary Figure 2

Supplementary Figure 3

Supplementary Figure 4

Supplementary Figure 5

Supplementary Figure 6

Supplementary Figure 7

Supplementary Figure 8

Supplementary Figure 9

Supplementary Figure Legends

Supplementary file 1

Reproducibility checklist

author contribution form

## Data Availability

The published article includes all data sets generated/analyzed for this study.
