# Supplementary figures and images for: YTHDF1 promotes breast cancer cell growth, DNA damage repair and chemoresistance
Source: Cell Death Dis. 2022 Mar 12;13(3):230. doi: 10.1038/s41419-022-04672-5 (PMC8918344; doi:10.1038/s41419-022-04672-5)

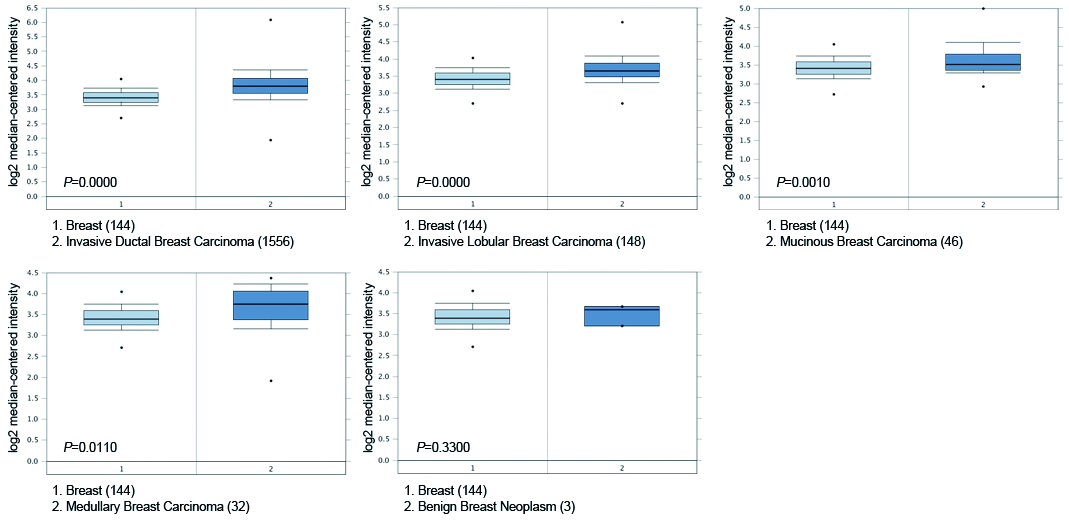

Supplement: Supplementary file 1 — Supplementary Figure 1 [file 41419_2022_4672_MOESM1_ESM.tif]

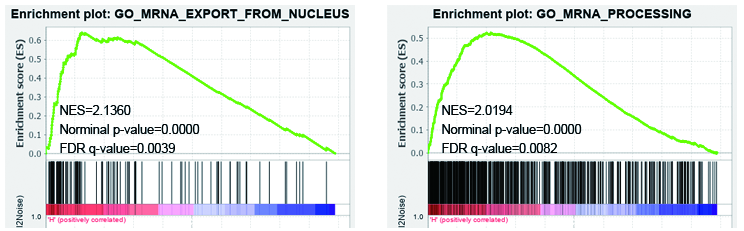

Supplement: Supplementary file 2 — Supplementary Figure 2 [file 41419_2022_4672_MOESM2_ESM.tif]

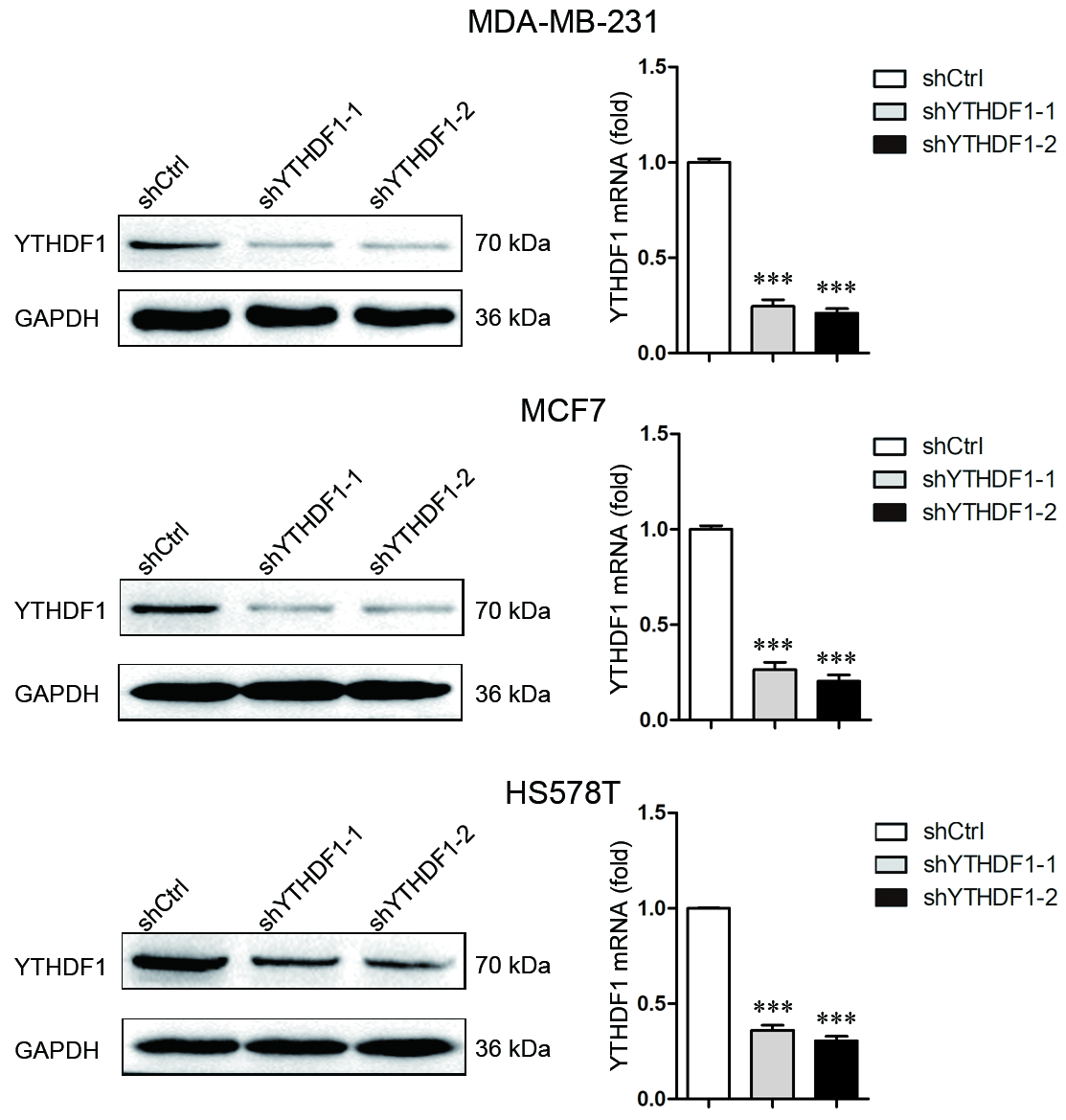

Supplement: Supplementary file 3 — Supplementary Figure 3 [file 41419_2022_4672_MOESM3_ESM.tif]

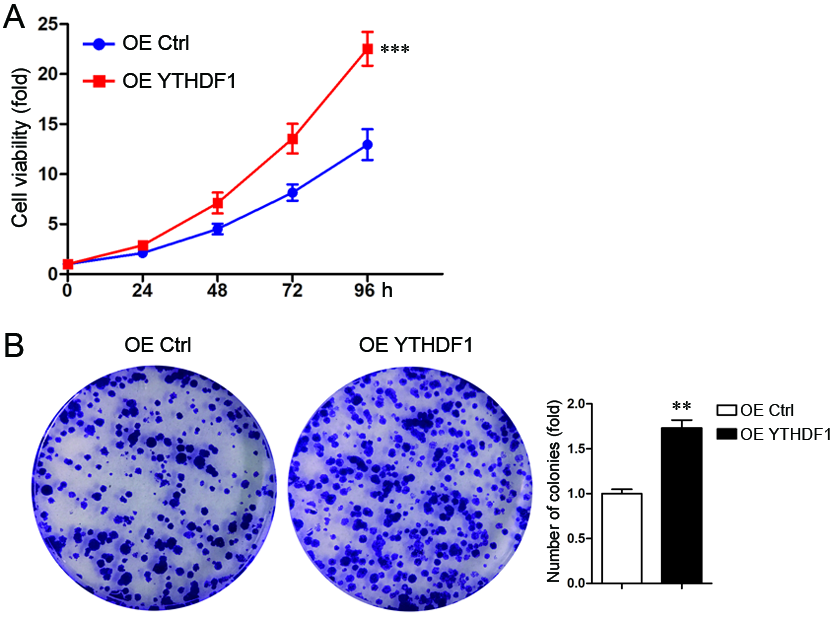

Supplement: Supplementary file 4 — Supplementary Figure 4 [file 41419_2022_4672_MOESM4_ESM.tif]

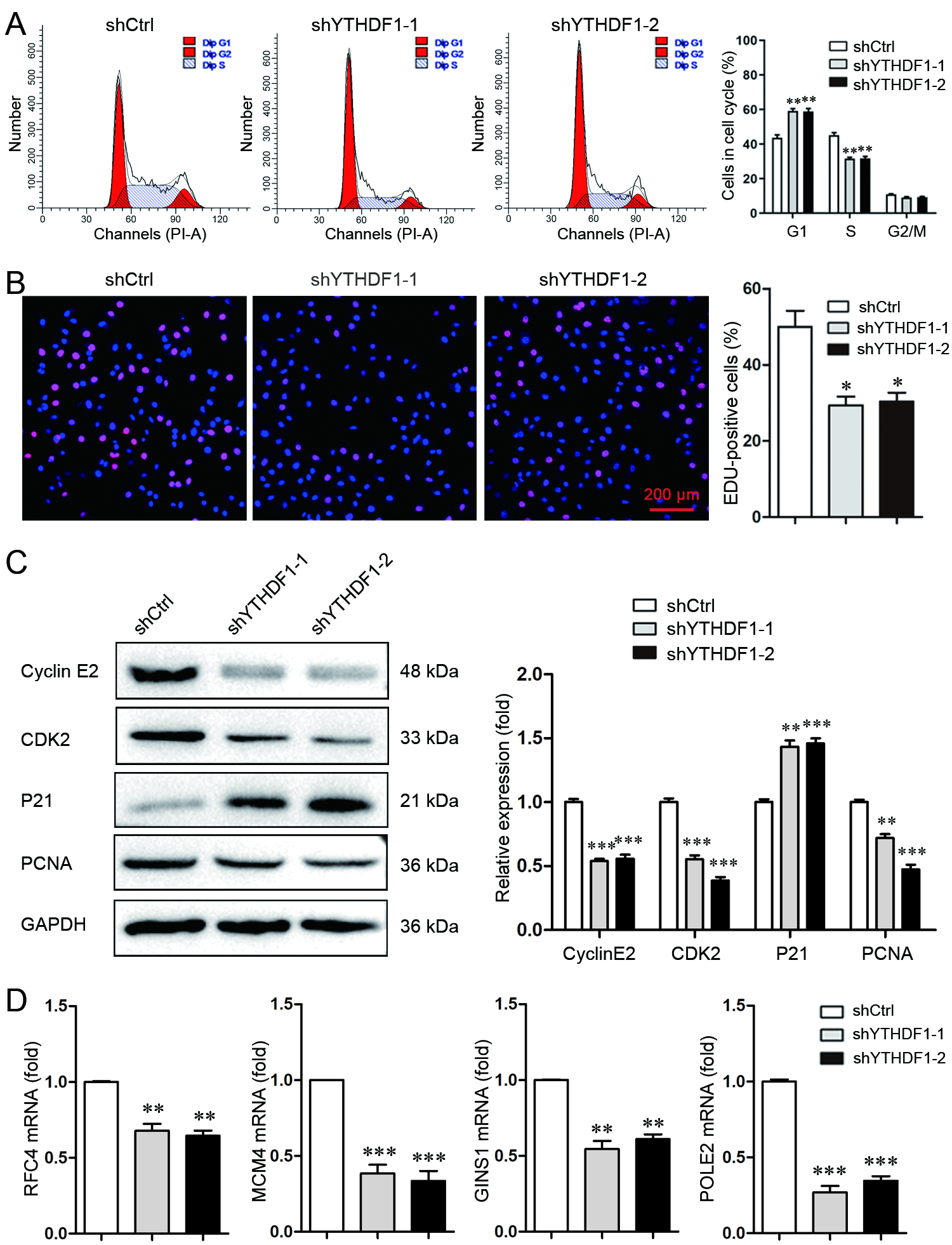

Supplement: Supplementary file 5 — Supplementary Figure 5 [file 41419_2022_4672_MOESM5_ESM.tif]

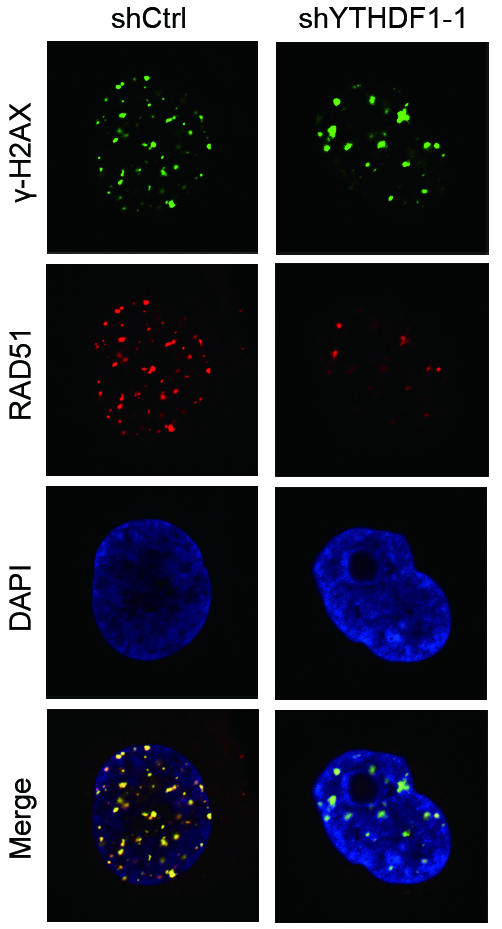

Supplement: Supplementary file 6 — Supplementary Figure 6 [file 41419_2022_4672_MOESM6_ESM.tif]

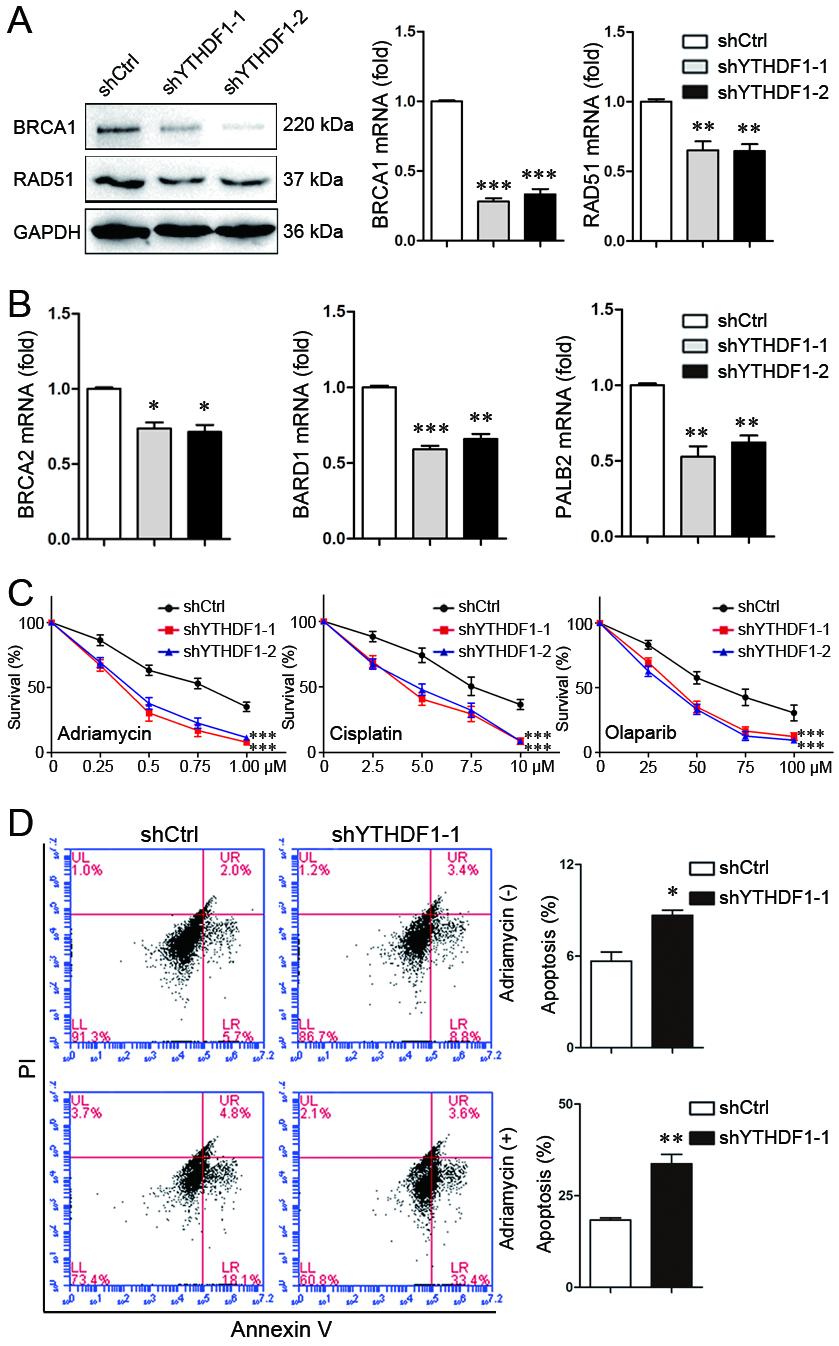

Supplement: Supplementary file 7 — Supplementary Figure 7 [file 41419_2022_4672_MOESM7_ESM.tif]

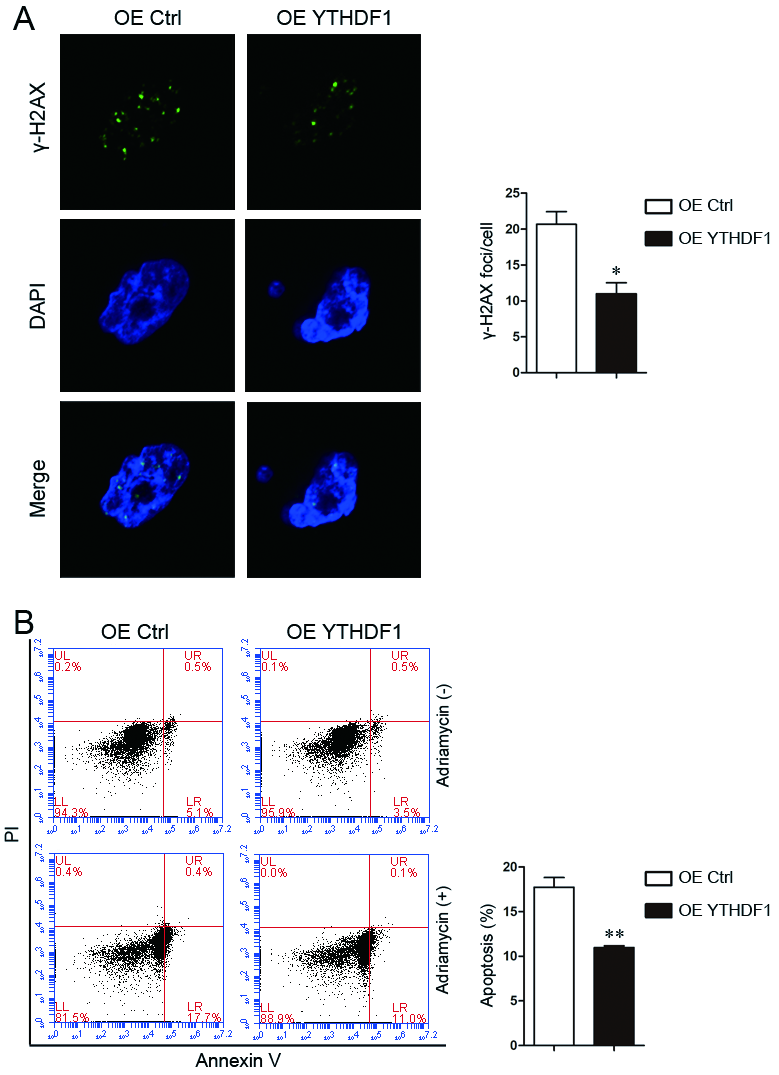

Supplement: Supplementary file 8 — Supplementary Figure 8 [file 41419_2022_4672_MOESM8_ESM.tif]

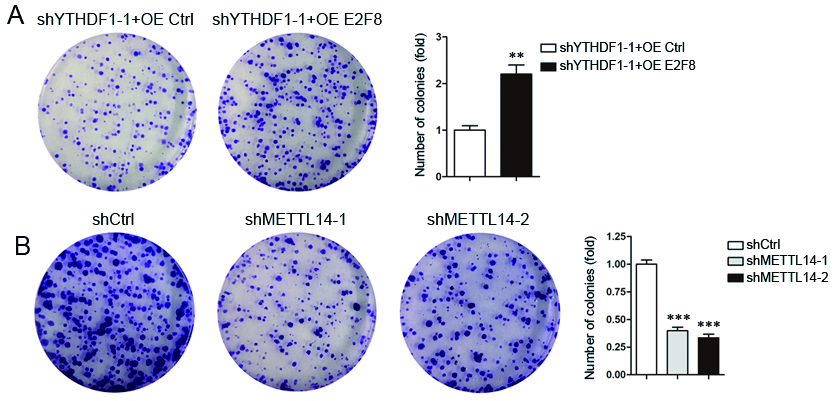

Supplement: Supplementary file 9 — Supplementary Figure 9 [file 41419_2022_4672_MOESM9_ESM.tif]
