# Supplementary material for: YTHDF1 promotes breast cancer cell growth, DNA damage repair and chemoresistance
Source: Cell Death Dis. 2022 Mar 12;13(3):230. doi: 10.1038/s41419-022-04672-5 (PMC8918344; doi:10.1038/s41419-022-04672-5)
Supplement: Supplementary file 10 — Supplementary Figure Legends [file 41419_2022_4672_MOESM10_ESM.docx]

**Supplementary Figure 1.** **YTHDF1 expression in Curtis database.** YTHDF1 expression in Curtis database was compared between breast tumors and normal mammary tissues in Oncomine.

**Supplementary Figure 2. GSEA in TCGA database.** YTHDF1-related enrichment plots were shown.

**Supplementary Figure 3. YTHDF1 knockdown in breast cancer cells.** YTHDF1 expression was detected by Western blot and Real-time PCR in MDA-MB-231, MCF7 and HS578T cells. Mean±SD, n=3. ****P*<0.001, *vs* shCtrl.

**Supplementary Figure 4. Promotion of cell growth by YTHDF1. (A)** Viability of MDA-MB-231 cells was analyzed by CCK8. Mean±SD, n=3. ****P*<0.001, *vs* OE Ctrl. (B) Growth of MDA-MB-231 cells was determined by Colony formation. Mean±SD, n=3. **P*<0.05, *vs* OE Ctrl.

**Supplementary Figure 5. Promotion of S-phase entry and DNA replication by YTHDF1.** (A) Cell cycle of MCF7 cells was analyzed by Flowcytometry. Mean±SD, n=3. ***P*<0.01, *vs* shCtrl. (B) DNA replication of MCF7 cells was determined by EDU staining. Scale bar: 200μm. Mean±SD, n=3. **P*<0.05, *vs* shCtrl. (C) Cyclin E2, CDK2, P21 and PCNA expression in MCF7 cells was detected by Western blot. Mean±SD, n=3. ***P*<0.01, ****P*<0.001, *vs* shCtrl. (D) RFC4, MCM4, GINS1 and POLE2 expression in MCF7 cells was detected by Real-time PCR. Mean±SD, n=3. ***P*<0.01, ****P*<0.001, *vs* shCtrl.

**Supplementary Figure 6. Induction of RAD51 recruitment to DNA damage site by YTHDF1.** γ-H2AX and RAD51 foci formation in MDA-MB-231 cells was detected by Immunofluorescence 2 hours after treatment with Adriamycin (300nM).

**Supplementary Figure 7. Enhancement of DNA damage repair and chemoresistance by YTHDF1.** (A) BRCA1 and RAD51 expression in MCF7 cells was detected by Western blot and Real-time PCR. Mean±SD, n=3. ***P*<0.01, ****P*<0.001, *vs* shCtrl. (B) BRCA2, BARD1 and PALB2 expression in MCF7 cells was detected by Real-time PCR. Mean±SD, n=3. **P*<0.01, ***P*<0.05, ****P*<0.001, *vs* shCtrl. (C) Viability of MCF7 cells was analyzed by CCK8 48 hours after treatment with different concentrations of Cisplatin or Olaparib. Mean±SD, n=3. ****P*<0.001, *vs* shCtrl. (D) Death of MCF7 cells was detected by Flowcytometry 24 hours after treatment with or without Adriamycin (300nM). Mean±SD, n=3. **P*<0.01, ***P*<0.05, *vs* shCtrl.

**Supplementary Figure 8. Promotion of DNA damage repair and chemoresistance by YTHDF1.** (A) γ-H2AX foci formation in MDA-MB-231 cells was detected by Immunofluorescence 12 hours after treatment with Adriamycin (300nM). Mean±SD, n=3. **P*<0.05, *vs* OE Ctrl. (B) Death of MDA-MB-231 cells was detected by Flowcytometry 24 hours after treatment with or without Adriamycin (300nM). Mean±SD, n=3. ***P*<0.01, *vs* OE Ctrl.

**Supplementary Figure 9. Colony formation analysis. (A)** Growth of MDA-MB-231 cells was determined by Colony formation. Mean±SD, n=3. ***P*<0.01, *vs* shYTHDF1-1+OE Ctrl. (B) Growth of MDA-MB-231 cells was determined by Colony formation. Mean±SD, n=3. ****P*<0.001, *vs* shCtrl.
