# Supplementary material for: YTHDF1 promotes breast cancer cell growth, DNA damage repair and chemoresistance
Source: Cell Death Dis. 2022 Mar 12;13(3):230. doi: 10.1038/s41419-022-04672-5 (PMC8918344; doi:10.1038/s41419-022-04672-5)

Supplementary Fig. 3

YTHDF1 (MDA-MB-231) GAPDH (MDA-MB-231)


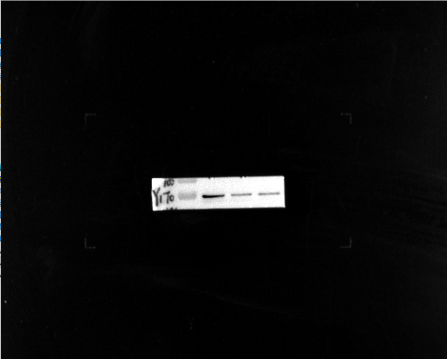

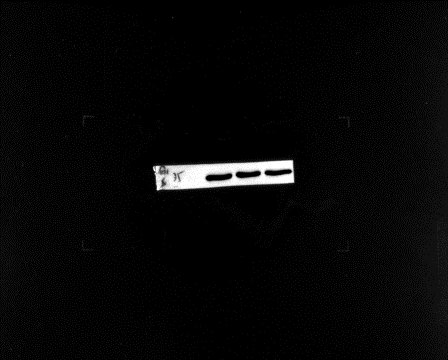


YTHDF1 (MCF7) GAPDH (MCF7)


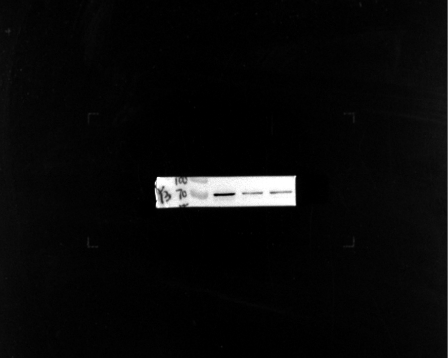

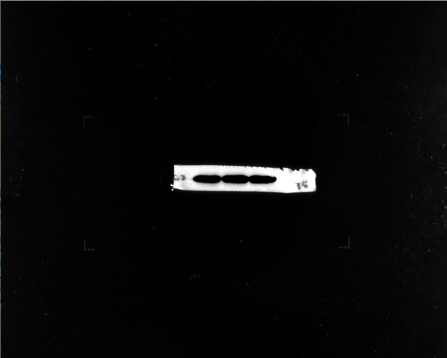


YTHDF1 (HS578T) GAPDH (HS578T)


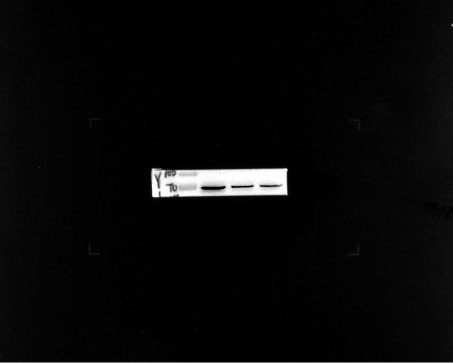

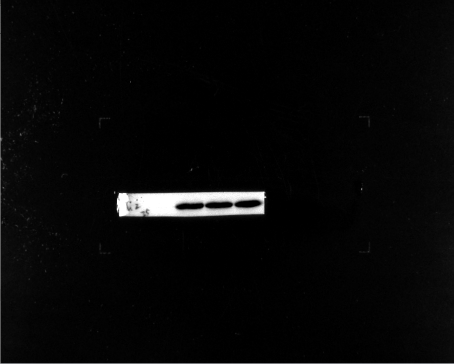


Fig. 3 C

Cyclin E2 CDK2


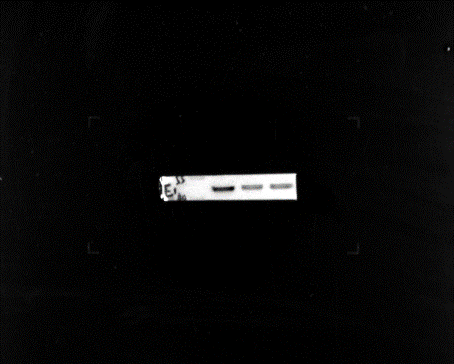

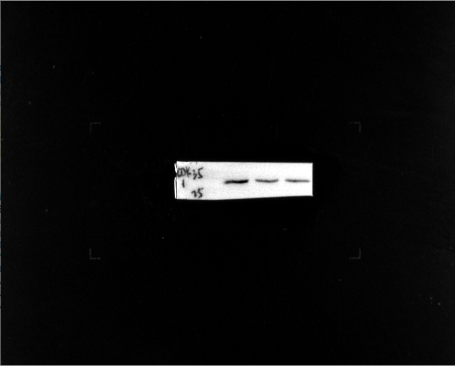


P21 PCNA


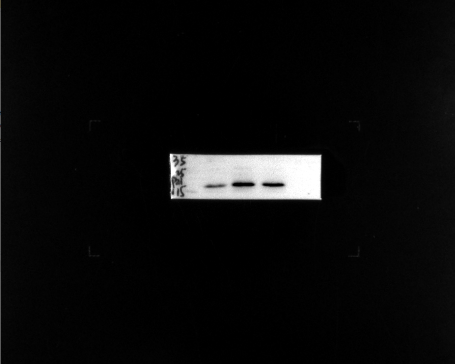

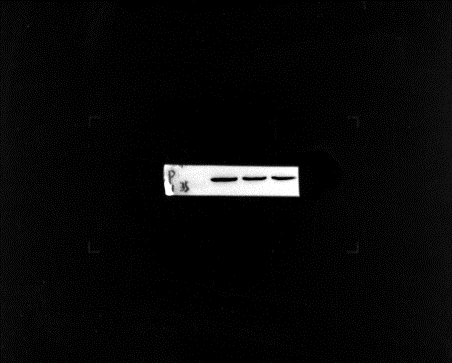


GAPDH


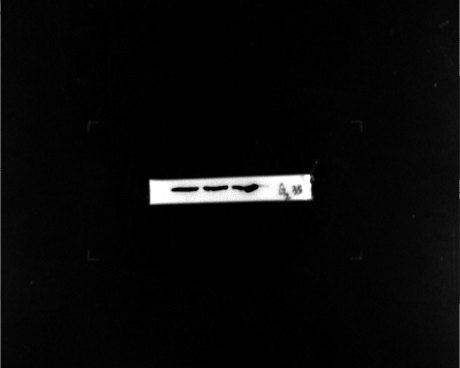


Supplementary Fig. 5C

Cyclin E2 CDK2


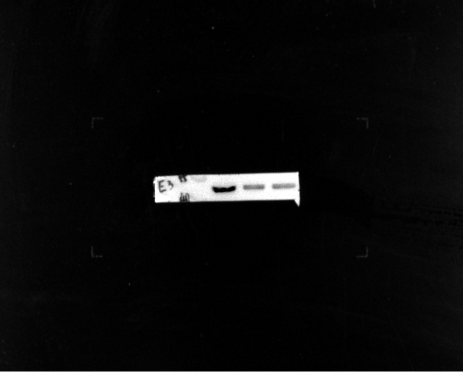

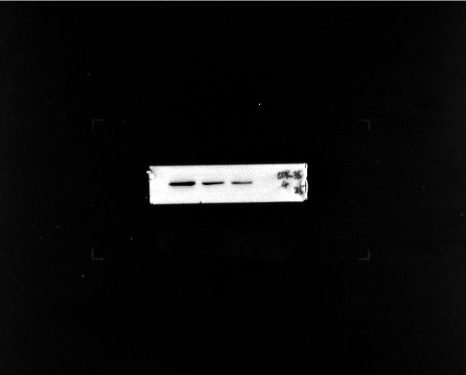


P21 PCNA


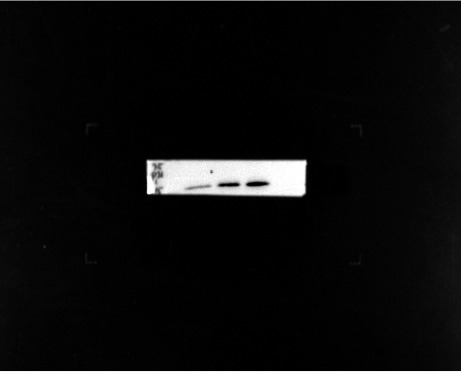

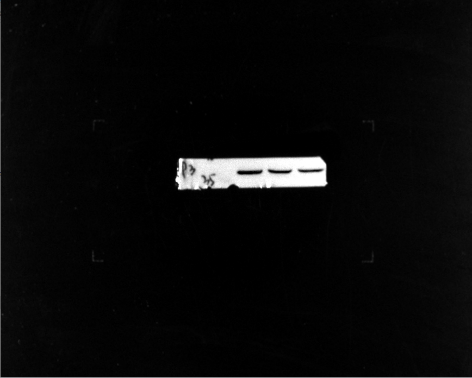


GAPDH


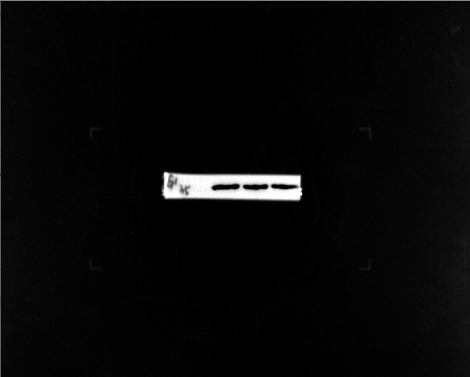


Fig. 4A

BRCA1 RAD51


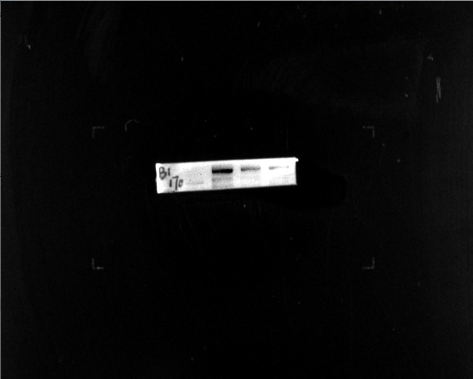

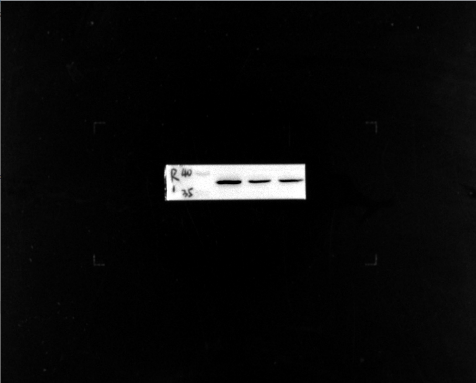


GAPDH


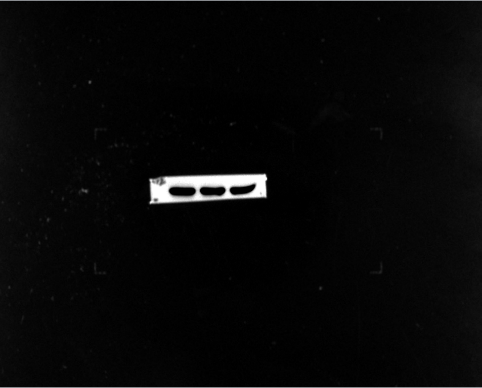


Supplementary Fig. 7A

BRCA1 RAD51


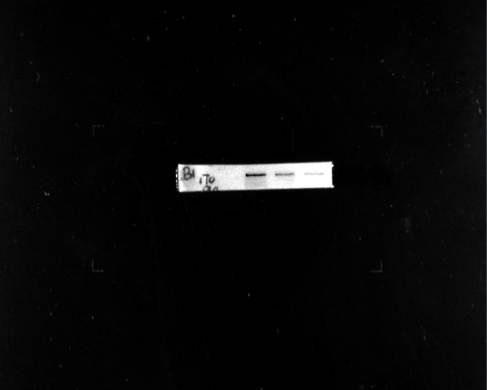

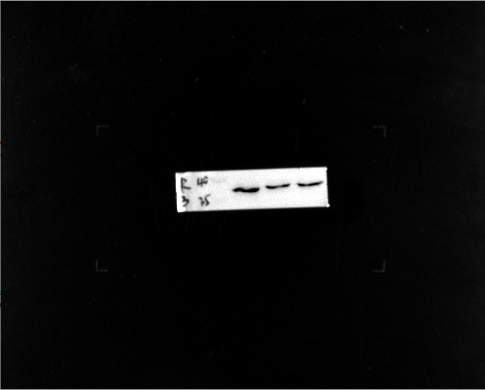


GAPDH


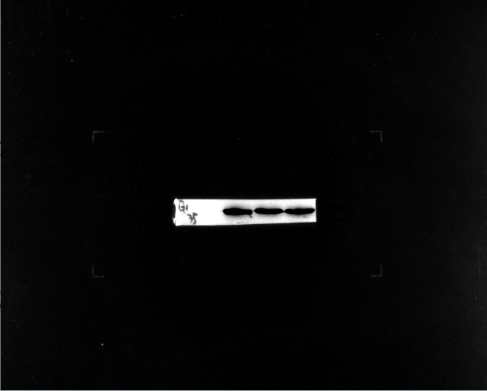


Fig.5C

E2F8 GAPDH


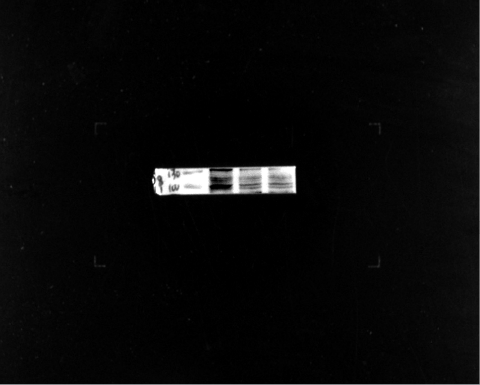

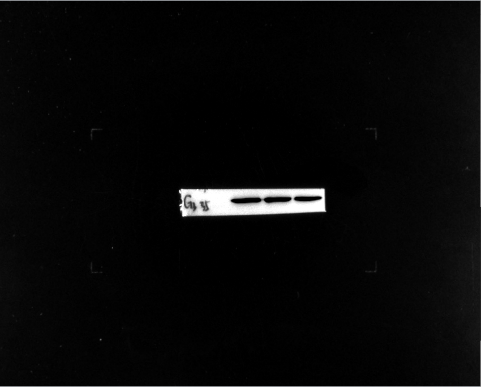


Fig.5D

E2F8 GAPDH


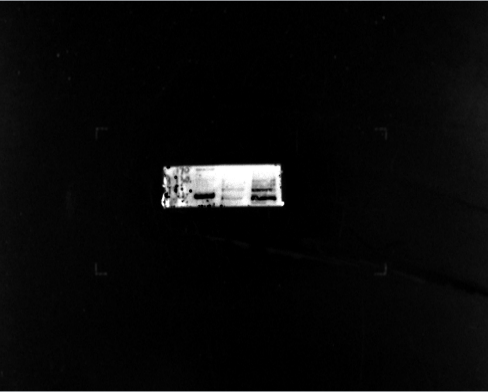

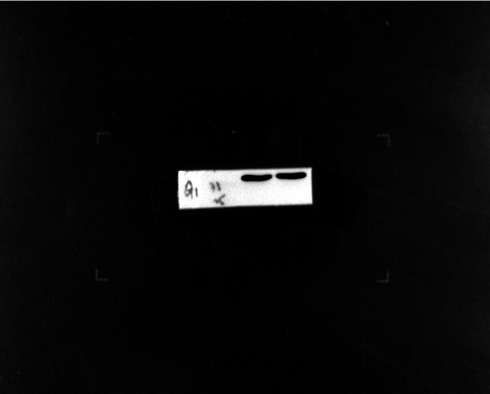


Fig.6C

METTL14 (MDA-MB-231) GAPDH (MDA-MB-231)


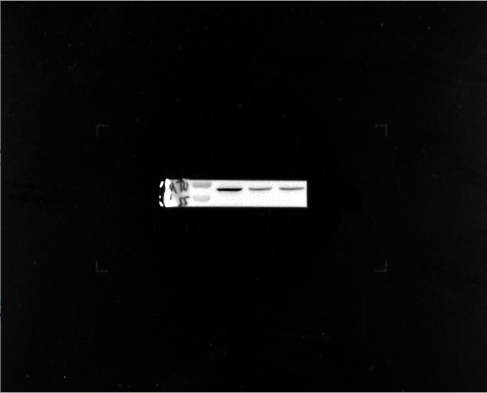

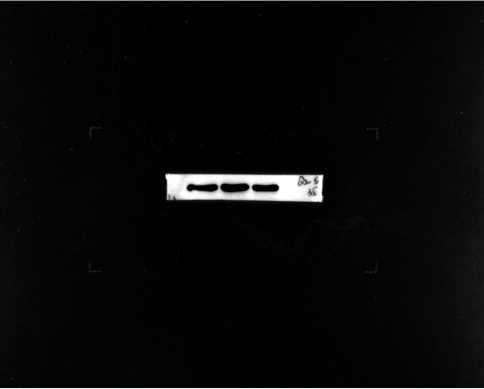


METTL14 (MCF7) GAPDH (MCF7)


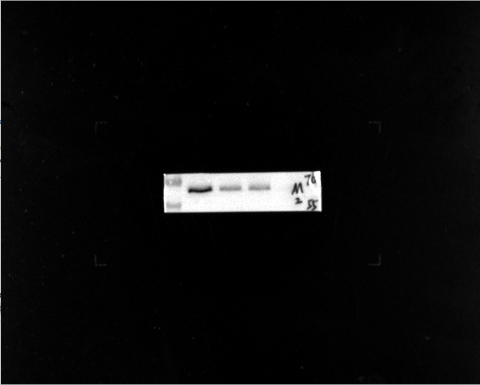

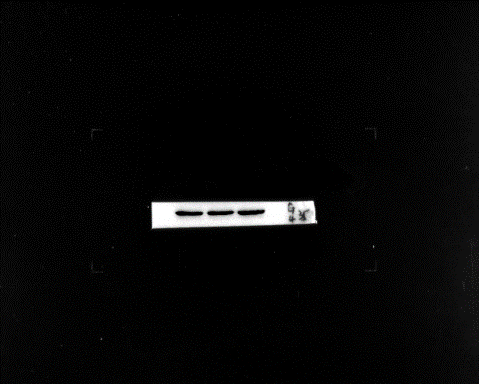


Fig.6F

E2F8 GAPDH


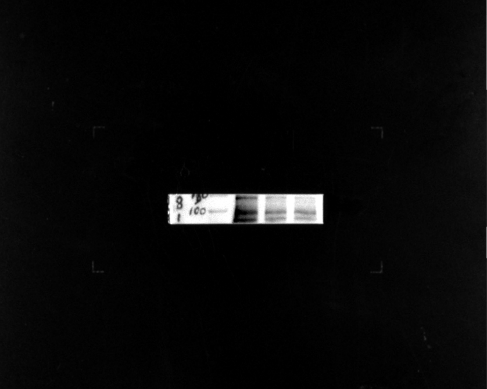

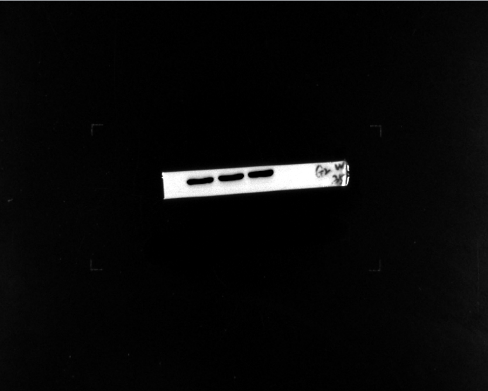

Supplement: Supplementary file 11 — Supplementary file 1 [file 41419_2022_4672_MOESM11_ESM.docx]
