# Supplementary material for: YTHDF1 promotes breast cancer cell growth, DNA damage repair and chemoresistance
Source: Cell Death Dis. 2022 Mar 12;13(3):230. doi: 10.1038/s41419-022-04672-5 (PMC8918344; doi:10.1038/s41419-022-04672-5)
Supplement: Supplementary file 13 — author contribution form [file 41419_2022_4672_MOESM13_ESM.pdf]

DECLARATION OF CONTRIBUTIONS TO ARTICLE

ADMC

Manuscript Number: CDDIS-21-3832

Journal Name: *Cell Death & Disease* (the 'Journal')

Proposed Title of the Contribution:

YTHDF1 promotes breast cancer cell growth, DNA damage repair and chemoresistance (the 'Contribution)

Author(s):

Yu Sun, Dan Dong, Yuhong Xia, Liying Hao, Wei Wang, and Chenghai Zhao (the 'Authors')

For all *CDDis* articles, each person named as an author in the published version must be able to show he or she has contributed substantially to the article.

Authorship credit should be based on 1) substantial contributions to conception and design, acquisition of data, or analysis and interpretation of data; 2) drafting the article or revising it critically for important intellectual content; and 3) final approval of the version to be published. Authors should meet conditions 1, 2 and 3.

Any person who cannot be shown to have made a substantial contribution to the article cannot be listed as an author in the final version. The name of any person who is deemed to have made a minor contribution can, however, appear in the Acknowledgments section of the article.

Please complete the table below to indicate the contributions of all named authors to the manuscript.

Author Full Name:	Specification of Contribution to the Manuscript:
Yu Sun	performed most experiments and statistical analyses
Dan Dong	performed part of experiments and statistical analyses
Yuhong Xia	performed part of experiments and statistical analyses
Liying Hao	conceived, designed and supervised the study
Wei Wang	conceived, designed and supervised the study
Chenghai Zhao	conceived, designed and supervised the study

Please complete the table below to indicate the contributions of all named authors to the figures.

Figure 1:

Yu Sun performed the in silico analysis.

Figure 2:

Yu Sun performed the CCK8, immunohistochemistry staining, western blot, clone and tumorisphere formation. Yu Sun and Dan Dong performed the in vivo animal experiment.

Figure 3:

Yu Sun performed the flow cytometry and western blot. Yu Sun, Dan Dong and Yuhong Xia performed the EDU assay and Real-time PCR.

Figure 4:

Yu Sun and performed the western blot, immunofluorescence, CCK8. Yu Sun and Yuhong Xia performed the Real-time PCR and the apoptosis assay.

Figure 5:

Yu Sun performed the in silico analysis. Yu Sun performed the western blot, Real-time PCR and immunofluorescence. Yu Sun, Dan Dong and Yuhong Xia performed the EDU assay.

Figure 6:

Yu Sun performed the western blot. Yu Sun, Dan Dong and Yuhong Xia performed the Chip assay and mRNA stability assay.

Signed for and on behalf of the Author(s):	Print Name:	Date:
Chenghai Zhao	Chenghai Zhao	2.16. 2022
